# An international survey in Latin America on the practice of interventional cardiology during the COVID-19 pandemic, with a particular focus on myocardial infarction

**DOI:** 10.1007/s12471-020-01440-y

**Published:** 2020-06-30

**Authors:** J. Mayol, C. Artucio, I. Batista, A. Puentes, J. Villegas, R Quizpe, V. Rojas, J. Mangione, J. Belardi, STEMI Working Group of Stent-Save a Life! LATAM and SOLACI (Latin American Society of Interventional Cardiology), Jorge Mayol, Carolina Artucio, Ignacio Batista, Angel Puentes, John Gough, Luis Urna, Jorge Villegas, Luis Gutiérrez Jaikel, Ronald Aroche, Ricardo Quizpe, Marco Fuentes, Hector Mora, Francisco Somoza, Patricio Ortiz, Daniel Meneses, Alfaro Marchena, Victor Rojas, Cesar Conde, Aramis Gomez, Pedro Hidalgo, Jose Mangione, Jorge Belardi

**Affiliations:** 1 Centro Cardiológico Americano, Montevideo, Uruguay; 2 Hemodinamia del Litoral, Salto, Uruguay; 3 Instituto de Cardiología Intervencionista de Casa de Galicia, Montevideo, Uruguay; 4 Servicio de Hemodinamia y Cardiología Intervencionista del Hospital Central de las FF.AA, Montevideo, Uruguay; 5 Hospital San Juan de Dios, Santiago de Chile, Chile; 6 Clínica San Rafael, Bogotá, Colombia; 7 Hospital Santa Inés, Cuenca, Ecuador; 8 Hospital José Carrasco Arteaga, Cuenca, Ecuador; 9 Hospital de Clínicas, Asunción, Paraguay; 10 Hospital Beneficência Portuguesa, Sao Pablo, Brazil; 11 ICBA, Buenos Aires, Argentina

**Keywords:** ST-elevation myocardial infarction, COVID-19, Telematic survey, Percutaneous coronary intervention

## Abstract

**Introduction:**

A reduction in the number of interventional cardiology procedures has emerged as a result of the COVID-19 pandemic. A survey was performed to quantify this decrease and the impact on the management of myocardial infarction in Latin America.

**Methods:**

A telematic survey was conducted for all countries in Latin America. Diagnostic catheterisations, coronary and structural interventions, as well as the incidence and delay to reperfusion therapy of myocardial infarction (STEMI), were recorded. Two periods were compared: from 24 February to 8 March 2020 (pre-COVID-19) and another 2‑week period that varied according to country (COVID-19).

**Results:**

Responses were obtained from 79 centres in 20 countries. There was a significant decrease in the number of diagnostic procedures (−65.2%), coronary interventions (−59.4%), structural therapeutics (−86.1%) and STEMI care (−51.2%). A decrease was noted in the incidence of STEMI, but also a delay in the time to STEMI reperfusion. While there was a variation in activity in interventional cardiology between countries, patient behaviour was rather homogeneous.

**Conclusions:**

A significant reduction in healthcare activity has been noted during the COVID-19 pandemic, including STEMI care, with the risk of increased mortality and/or morbidity following STEMI. Healthcare providers should encourage patients with suspected symptoms of STEMI to call for emergency care to ensure rapid diagnosis and timely reperfusion treatment.

**Electronic supplementary material:**

The online version of this article (10.1007/s12471-020-01440-y) contains supplementary material which is available to authorized users.

## What’s new?

- A marked reduction in interventional cardiology activity has been observed in Latin America during the COVID-19 pandemic, both in elective and emergency procedures, particularly in patients with STEMI.
- In Latin America patient behaviour has been quite homogeneous, although varying quarantine measures in the individual countries have restricted mobility to different degrees.
- The health authorities should be alert regarding the care of STEMI patients during the COVID-19 pandemic.

## Introduction

The coronavirus disease 2019 (COVID-19) pandemic has led to the installation of unprecedented health measures in almost every country worldwide. In some countries, particularly in Europe, healthcare systems have become saturated, especially emergency departments and intensive care units, potentially influencing healthcare availability for patients with acute coronary syndromes (ACS). The prognosis in patients with ST-segment elevation myocardial infarction (STEMI) is particularly dependent on rapid diagnosis and prompt implementation of reperfusion therapy [1].

However, the necessary quarantine measures have reduced people’s mobility and appear to have affected particularly the number of high-risk patients consulting medical services and the delay in doing so. This new reality has recently been objectified in countries in Asia, Europe and North America, with fewer interventional cardiology procedures being performed during the pandemic [2–4].

In Latin America, the first COVID-19 cases were established later than in Asia and Europe, potentially altering patients’ demographics. The impact of this pandemic on healthcare provision for non-COVID-19 patients in Latin America has not yet been assessed, especially for those with ACS. Cardiovascular disease has a high prevalence in Latin America, where it represents the leading cause of death, underlining the significance of this problem. Therefore, it is important to gather information about the impact of the COVID-19 pandemic on interventional cardiology activity in Latin America.

For 3 years, the Stent-Save a Life initiative! (S-SL!) has been working in Latin America with the endorsement of the Latin American Society of Interventional Cardiology (SOLACI), promoting the application of clinical guidelines on the care of STEMI patients, stimulating more and better reperfusion treatment, both through academic activities and the organisation of regional reperfusion networks. The presence of this Latin American network was used to evaluate the effect of the COVID-19 pandemic on STEMI care. A telematic survey was performed with the main objective of quantifying the degree of variation in care activity in interventional cardiology services due to the COVID-19 pandemic, with particular attention to STEMI. A secondary objective was to analyse the changes in the diagnosis and treatment of acute myocardial infarction with ST-segment elevation.

## Methods

A cross-cutting, descriptive and observational study was conducted by means of an opinion survey to assess activity in Latin American interventional cardiology centres during one fortnight before and a fortnight after the introduction of quarantine or social isolation measures in each country in response to the COVID-19 pandemic.

This survey consisted of three blocks of questions:

1. In the first block, the respondent was characterised by requesting: country, city, name of the centre, head of the centre, person responsible for answering the survey questions, and contact mail.
2. The second block quantified the procedures: total number of coronary angiography (CAG) procedures, CAG performed in patients with ACS, total number of percutaneous coronary interventions (PCI), PCI for STEMI and structural interventions, before and after the introduction of quarantine measures as a result of the pandemic in the respective countries. The variation between the two periods, expressed as a percentage, was calculated. An analysis was carried out per country and for the whole of Latin America.
3. The third block presented closed questions with multiple answers, aimed at the analysis of the diagnosis and treatment of STEMI.

Included in this web survey were all interventional cardiology centres of Latin American countries whose members are in the SOLACI database, which were invited to participate by mail. The data used came from the local databases of each participating centre. The Google Forms tool was used, self-adjusting to multi-platforms.

The requested data corresponded to two periods of 14 days each, separated by an interval of 2 weeks. These periods were defined individually for each of the 20 Latin American countries, according to the date of declaration of the health emergency or introduction of quarantine measures in each of them.

The pre-COVID-19 period was the same in all cases, 24 February 2020 to 8 March 2020. The COVID-19 period was defined as shown in Table 1, confirming the dates with references from each country.

**Table 1:** Definition of pre-COVID-19 and COVID-19 periods by region and country of Latin America

Region	Country	Quarantine date	Pre-COVID-19 period	COVID-19 period
Central America and the Caribbean	Belize	11 April 2020	24 February–8 March 2020	30 March–12 April 2020
	Costa Rica	17 March 2020	24 February–8 March 2020	23 March–5 April 2020
	Cuba	20 March 2020	24 February–8 March 2020	30 March–12 April 2020
	Dominican Republic	16 March 2020	24 February–8 March 2020	23 March–5 April 2020
	El Salvador	23 March 2020	24 February–8 March 2020	30 March–12 April 2020
	Guatemala	22 March 2020	24 February–8 March 2020	30 March–12 April 2020
	Honduras	16 March 2020	24 February–8 March 2020	23 March–5 April 2020
	Mexico	30 March 2020	24 February–8 March 2020	30 March–12 April 2020
	Nicaragua	–	24 February–8 March 2020	30 March–12 April 2020
	Panama	25 March 2020	24 February–8 March 2020	30 March–12 April 2020
Andean	Colombia	24 March 2020	24 February–8 March 2020	30 March–12 April 2020
	Ecuador	12 March 2020	24 February–8 March 2020	23 March–5 April 2020
	Peru	16 March 2020	24 February–8 March 2020	23 March–5 April 2020
	Venezuela	16 March 2020	24 February–8 March 2020	23 March–5 April 2020
Southern Cone	Argentina	19 March 2020	24 February–8 March 2020	23 March–5 April 2020
	Bolivia	16 March 2020	24 February–8 March 2020	23 March–5 April 2020
	Brazil	21 March 2020	24 February–8 March 2020	23 March–5 April 2020
	Chile	26 March 2020	24 February–8 March 2020	30 March–12 April 2020
	Paraguay	10 March 2020	24 February–8 March 2020	23 March–5 April 2020
	Uruguay	14 March 2020	24 February–8 March 2020	23 March–5 April 2020

**Fig. 1:**
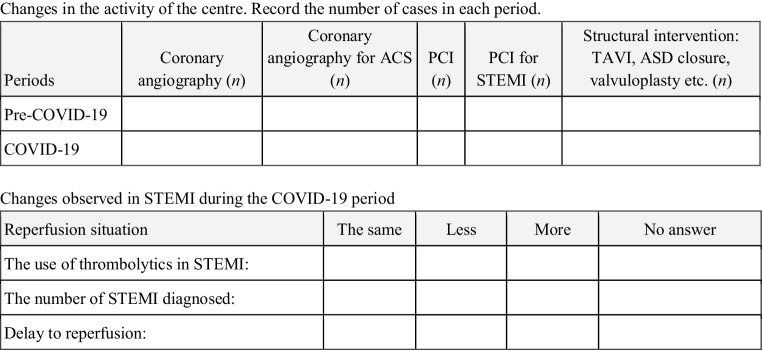
Form requesting information in the international survey in Latin America on the practice of interventional cardiology during the COVID-19 pandemic. *ACS* acute coronary syndrome, *STEMI* ST-segment elevation myocardial infarction, *PCI* percutaneous coronary intervention, *TAVI* percutaneous aortic valve implantation, *ASD* atrial septal defect

The survey was sent on 17 April 2020, with responses being received by 30 April 2020; the information requested is summarised in Fig 1.

## Results

Information was received from 79 centres in the 20 Latin American countries consulted (Table 2). The participating cardiology centres are detailed in Appendix 2 (see Electronic Supplementary Material).

**Table 2:** Variation in the number of procedures per country and for the whole of Latin America

		A	All procedures			B	Coronary angiography			C	PCI			D	Structural interventions		
Country	No. of centres		Pre-COVID-19	COVID-19	Variation (%)		Pre-COVID-19	COVID-19	Variation (%)		Pre-COVID-19	COVID-19	Variation (%)		Pre-COVID-19	COVID-19	Variation (%)
*Argentina*	6		290	90	−68.9		206	62	−69.9		76	25	−67.1		8	3	−62.5
*Belize*	1		15	6	−60		11	6	−45.5		4	–	−100		–	–	–
*Bolivia*	2		37	10	−72.9		25	7	−72		12	3	−75		–	–	–
*Brazil*	7		855	337	−60.6		617	235	−61.9		204	100	−50.9		34	2	−94.1
*Chile*	14		1354	473	−65.1		895	295	−67		434	176	−59.4		25	2	−92
*Colombia*	9		954	305	−68		587	189	−67.8		325	100	−69.2		42	16	−61.9
*Costa Rica*	1		332	151	−54.5		220	100	−54.5		100	50	−50		12	1	−91.7
*Cuba*	1		42	–	−100		26	–	−100		16	–	−100		–	–	–
*Ecuador*	5		315	48	−84.8		184	24	−86.9		101	24	−76.2		30	–	−100
*El Salvador*	1		17	2	−88.2		10	1	−90		7	1	−85.7		–	–	–
*Guatemala*	2		66	9	−86.4		44	8	−81.8		13	1	−92.3		9	–	−100
*Honduras*	1		35	12	−65.7		25	6	−76		10	6	−40		–	–	–
*Mexico*	4		100	28	−72		47	16	−65.9		35	10	−97.1		18	2	−88.9
*Nicaragua*	1		38	6	−84.2		25	4	−84		13	2	−84.6		–	–	–
*Panama*	1		195	69	−64.6		128	44	−65.6		60	25	−58.3		7	–	−100
*Paraguay*	8		344	120	−65.1		222	75	−66.2		119	45	−62.2		3	–	−100
*Peru*	2		160	33	−79.4		100	19	−81		58	14	−75.9		2	–	−100
*Dominican Republic*	1		64	11	−82.8		38	4	−89.5		26	7	−73.1		–	–	–
*Uruguay*	8		456	311	−31.8		294	188	−36.1		153	122	−20.3		9	1	−88.9
*Venezuela*	4		34	27	−20.6		19	14	−26.3		13	12	−7.7		2	1	−50
*Total*	*79*		*5703*	*2048*	*−64.1*		*3723*	*1297*	*−65.2*		*1779*	*723*	*−59.4*		*201*	*28*	*−86.1*

*A* Variation in the total number of procedures per country and for the whole of Latin America. *B* Variation in the total number of coronary angiography procedures per country. *C* Variation in the total number of percutaneous coronary interventions (*PCI*) per country and for the whole of Latin America. *D* Variation in the number of structural interventions per country, including valvuloplasty, TAVI (percutaneous aortic valve implantation), atrial septal defect closure, etc.

During the pre-COVID-19 period, 5703 procedures were carried out in the centres in Latin America that responded to the survey. Of these, 3723 were CAG [1995 (53.6%) ACS], 1779 PCI [814 (45.8%) STEMI] and 201 structural interventions.

In the COVID-19 period, 2048 procedures were carried out in these centres. Of these, 1297 were CAG [884 (68.2%) ACS], 723 PCI [397 (54.9%) STEMI] and 28 structural interventions.

**Fig. 2:**
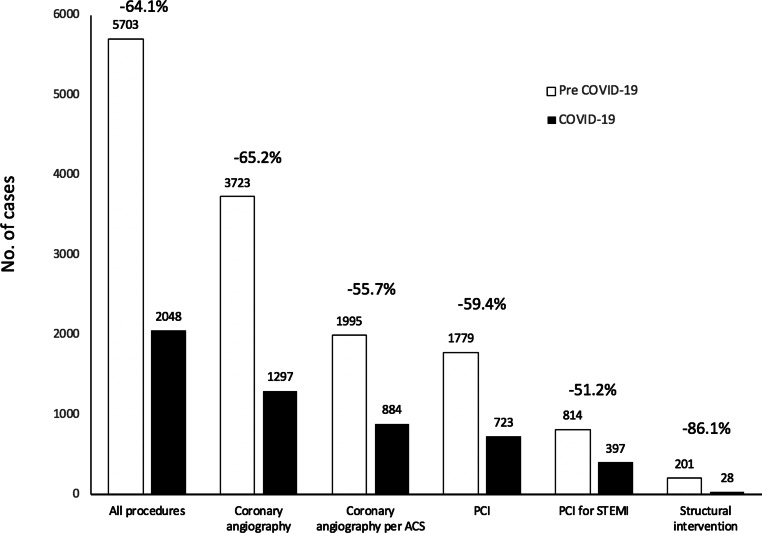
Variation in the different care activities in Latin America during the COVID-19 pandemic. *ACS* acute coronary syndrome, *STEMI* ST-segment elevation myocardial infarction, *PCI* percutaneous coronary intervention

All of the care activities showed a very significant decrease during the COVID-19 period, as depicted in Fig. 2. The total number of procedures decreased by 64.1%.

Table 2 (part A) shows the total number of procedures per country during the pre-COVID-19 and COVID-19 periods, as well as the variation, expressed as a percentage.

The total number of diagnostic procedures decreased by 65.2% (Table 2, part B), CAG for stable cardiovascular pathologies decreased by 76% (1728 pre-COVID-19 period vs 413 COVID-19 period) and CAG for ACS by 55.7% (Table 3, part A).

**Table 3:** Variation in the total number of procedures per country and for Latin America

	A	Coronary angiography for ACS			B	PCI for STEMI		
Country		Pre-COVID-19	COVID-19	Variation (%)		Pre-COVID-19	COVID-19	Variation (%)
*Argentina*		84	23	−72.6		21	12	−42.9
*Belize*		2	–	−100		2	–	−100
*Bolivia*		9	4	−55.6		5	1	−80
*Brazil*		286	152	−46.9		40	22	−45
*Chile*		400	219	−45.3		174	103	−40.8
*Colombia*		476	144	−69.7		166	72	−56.6
*Costa Rica*		150	50	−66.7		60	30	−40
*Cuba*		7	–	−100		2	–	−100
*Ecuador*		74	28	−62.2		53	21	−60.4
*El Salvador*		4	–	−100		2	–	–
*Guatemala*		15	8	−46.7		7	1	−85.7
*Honduras*		15	6	−60		10	3	−70
*Mexico*		32	14	−56.3		25	9	−64
*Nicaragua*		6	–	−100		5	–	−100
*Panama*		45	23	−48.9		35	23	−34.3
*Paraguay*		130	52	−60		92	41	−55.4
*Peru*		38	19	−50		21	11	−47.6
*Dominican Republic*		21	3	−85.7		19	–	−100
*Uruguay*		186	128	−31.2		67	42	−37.3
*Venezuela*		15	11	−26.7		8	6	−25
*Total*		*1995*	*884*	*−55.7*		*814*	*397*	*−51.2*

*A* Variation in the number of coronary angiography procedures for acute coronary syndromes (*ACS*) by country and for Latin America. *B* Variation in the number of percutaneous coronary interventions (*PCI*) for ST-elevation coronary infarction (*STEMI*) by country and for the whole of Latin America

Concerning the total number of PCI procedures, the variation was −59.4% (Table 2, part C), −51.2% for PCI in STEMI (Table 3, part B). The greatest variation (−86.1%) was observed in structural interventions (Table 2, part D).

**Fig. 3:**
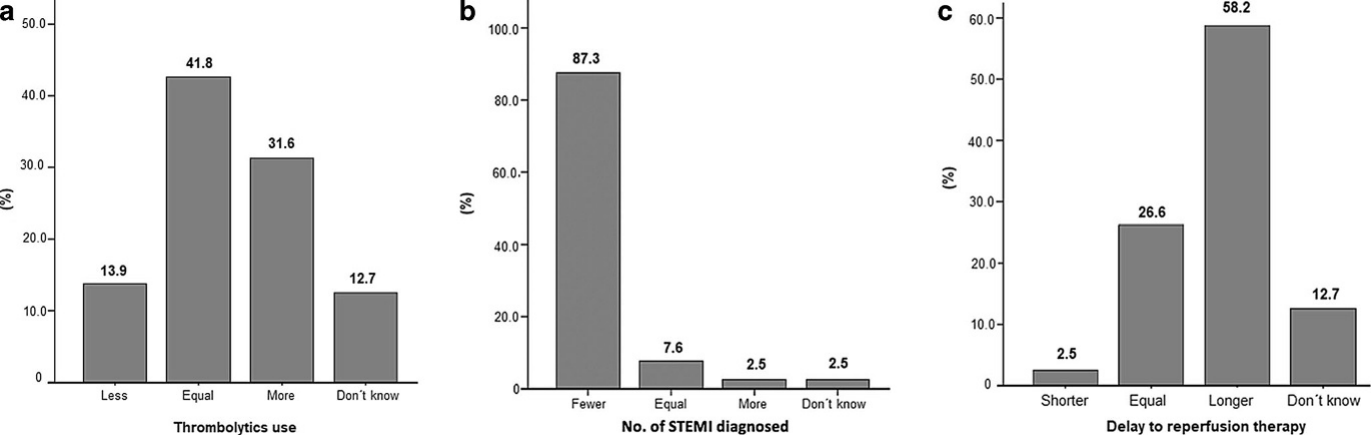
Changes observed in ST-segment elevation myocardial infarction (*STEMI*) during the COVID-19 period. **a** Use of thrombolytics for STEMI. **b** Number of STEMI diagnosed. **c** Delay to reperfusion treatment for STEMI

With regard to the outcome in STEMI patients, 42.5% of all respondents reported that the use of thrombolytics was the same in both periods (Fig. 3a); most (87.5%) considered that fewer cases of STEMI were diagnosed in the COVID-19 period (Fig. 3b) and more than half (58.8%) showed an increase in the length of the delay to reperfusion in STEMI patients (Fig. 3c).

## Discussion

The results show a clear and sustained decrease in all healthcare activity in the interventional cardiology centres in Latin America. The data are very homogeneous among the different countries, which expresses a common profile for the whole region.

This finding is consistent with recently published international studies [2–4]. A study from a single hospital in Hong Kong showed not only a sharp increase in time from symptom onset to first medical contact in STEMI patients treated after the infection control measures were instituted, but also delays in evaluating patients with STEMI after hospital arrival [2].

A Spanish study on the impact of the COVID-19 pandemic conducted by Rodríguez-Leor et al., with a special interest in the incidence of PCI for STEMI in 17 autonomous communities and 81 centres, showed a significant decrease in the number of diagnostic procedures (−57%), PCI (−48%), STEMI cases (−40%) and structural interventions (−81%) after quarantine [3].

Garcia et al. analysed and quantified the activity of nine high-volume primary PCI centres (i.e. those performing more than 100 primary PCI per year). A pre-COVID-19 period of 14 months (1 January 2019 to 29 February 2020) versus a COVID-19 period of 1 month (1–31 March 2020) was compared. Preliminary analyses in the early phase of the pandemic showed a reduction of 38% of the activation of the heart attack code [4].

Our study confirms these findings, showing that the non-urgent procedures were the ones that decreased most significantly. However, it is striking that procedures in ACS patients were also significantly reduced.

The primary goal of this study was to quantify the reduction in cardiac care during the COVID-19 period. It can be expected that the marked reduction in diagnostic and therapeutic procedures translates into an increase in cardiac mortality and morbidity. The clinical outcome of patients is most relevant and requires complex analysis, which was beyond the scope of this inventory study.

The study does not clarify the reasons for the decrease in cardiac interventions. It cannot be explained by the saturation of the health system, since at the time the survey was done, this phenomenon had been confirmed in a very isolated way, limited to particular cities. It is conceivable that confinement, which limited the mobility of people, as well as the fear of contagion from going to hospitals, constitute the background for the lower number of consultations, especially for those who suffer from ACS and are widely considered as one of the risk groups.

The homogeneity of the decrease in care activity contrasts with the different degrees of penetration or virulence of the infection in the 20 countries, as well as with the varied spectrum of confinement rigidity decreed by governments. There has been a mandatory quarantine declaration (Argentina), voluntary quarantine (Uruguay, Mexico, Chile), non-stringent recommendations (Brazil), and the no-quarantine declaration as in Nicaragua. Perhaps similar behaviour by different communities has prevailed over the other potential variables.

It is necessary to be aware of the situation during the evolution of this pandemic, to take the necessary measures, since the lack of adequate care is not only affecting the present situation but will also affect short- and mid-term outcomes because many patients have not received adequate care during the acute phase of coronary syndromes.

### Limitations

The survey has a selection bias, as it is a voluntary survey, with responses received from variable proportions of intervention centres in each country. While in countries such as Belize, Chile or Uruguay almost 100% of the centres were reached, in other countries, such as Brazil, Mexico or Argentina, fewer representative responses were obtained. In any case, all the Latin American countries that are members of SOLACI are represented. Data came from local databases in each centre, so it is possible that different criteria were applied during data collection.

Despite this, the number of procedures recorded is significant and the results are very homogeneous among the different countries, which expresses the same situation profile for the whole region.

## Conclusions

This study shows that in Latin America there has been a very significant decrease in care activity in interventional cardiology during the COVID-19 pandemic. This decrease has been predominantly in non-urgent patients, but it has also been very significant in those with ACS and especially those with STEMI. This finding shows that there may be a risk of increased mortality and/or morbidity from this pathology during the pandemic. Healthcare providers should encourage patients with suspected STEMI symptoms to contact emergency services promptly, to ensure rapid diagnosis and timely reperfusion treatment.

## Caption Electronic Supplementary Material

Appendix 1: STEMI Working Group of Stent-Save a Life! LATAM/SOLACI (Latin American Society of Interventional Cardiology)

Appendix 2: List of participating centres in the survey

## References

[1] Ibanez B (2018). 2017 ESC Guidelines for the management of acute myocardial in patients presenting with ST-segment elevation. Eur Heart J.

[2] Tam CF, Cheung KS, Lam S (2020). Impact of coronavirus disease 2019 (COVID-19) outbreak on ST-segment elevation myocardial infarction care in Hong-Kong, China. Cardiovasc Qual Outcomes.

[3] Rodríguez-Leor O (2020). Impact of the COVID-19 pandemic on healthcare activity in interventional cardiology in Spain. REC Interv Cardiol.

[4] Garcia S, Albaghdadi MS, Meraj PM (2020). Reduction in ST-segment elevation cardiac catheterization laboratory activations in the United States during COVID-19 pandemic. J Am Coll Cardiol.

