# Supplementary material for: An international survey in Latin America on the practice of interventional cardiology during the COVID-19 pandemic, with a particular focus on myocardial infarction
Source: Neth Heart J. 2020 Jun 30;28(7-8):424–30. doi: 10.1007/s12471-020-01440-y (PMC7325476; doi:10.1007/s12471-020-01440-y)
Supplement: Supplementary file 1 — Appendix 1: STEMI Working Group of Stent-Save a Life! LATAM/SOLACI (Latin American Society of Interventional Cardiology) [file 12471_2020_1440_MOESM1_ESM.docx]

### **Appendix 1** STEMI Working Group of Stent-Save a Life! LATAM/SOLACI (Latin American Society of Interventional Cardiology)

| **Name** | **Hospital** | **Country** |
| --- | --- | --- |
| Jorge Mayol | Centro Cardiológico Americano, Montevideo. SSL LATAM Regional Champion | Uruguay |
| Carolina Artucio | Instituto de Cardiología Intervencionista de Casa de Galicia, Montevideo. SSL Uruguay Project Manager | Uruguay |
| Ignacio Batista | Centro Cardiológico Americano, Montevideo. SSL Uruguay Country Manager | Uruguay |
| Angel Puentes | Hospital San Juan de Dios, Santiago de Chile | Chile |
| John Gough | Cardiology Centre Belize, Belize City | Belize |
| Luis Urna | Hospital San Juan de Dios, Santa Cruz de la Sierra | Bolivia |
| Jorge Villegas | Clínica San Rafael, Bogota | Colombia |
| Luis Gutiérrez Jaikel | Hospital Clínica Bíblica, San José | Costa Rica |
| Ronald Aroche | CIMEQ, Havana | Cuba |
| Ricardo Quizpe | Hospital Santa Inés, Cuenca | Ecuador |
| Marco Fuentes | Clínica Circulación, San Salvador | El Salvador |
| Hector Mora | Unidad de Cirugía Cardiovascular de Guatemala (UNICAR) | Guatemala |
| Francisco Somoza | Hospital CemeSa, San Pedro Sula | Honduras |
| Patricio Ortiz | Instituto Nacional de Cardiología ‘Ignacio Chávez’, Mexico City | Mexico |
| Daniel Meneses | Centro Nacional de Cardiología, Managua | Nicaragua |
| Alfaro Marchena | Hospital Arnulfo Arias Madrid, Panama | Panama |
| Victor Rojas | Hospital de Clínicas, Asunción | Paraguay |
| Cesar Conde | Instituto Nacional Cardiovascular, INCOR, Essalud, Lima | Peru |
| Aramis Gomez | Centro de Intervenciones Cardiovasculares, Santiago de los Caballeros | Dominican Republic |
| Pedro Hidalgo | Policlínica Amado, Maracaibo | Venezuela |
| Jose Mangione | Hospital BP – Unidade Paulista, Sao Paulo. SOLACI President | Brazil |
| Jorge Belardi | Instituto Cardiovascular de Buenos Aires, ICBA. SSL LATAM Region Champion | Argentina |
