# Supplementary material for: An international survey in Latin America on the practice of interventional cardiology during the COVID-19 pandemic, with a particular focus on myocardial infarction
Source: Neth Heart J. 2020 Jun 30;28(7-8):424–30. doi: 10.1007/s12471-020-01440-y (PMC7325476; doi:10.1007/s12471-020-01440-y)
Supplement: Supplementary file 2 — Appendix 2: List of participating centres in the survey [file 12471_2020_1440_MOESM2_ESM.docx]

### **Appendix 2** Participating centres

| **Country** | **City** | **Cardiology centre** | **Head of centre** | **Responsible for survey** |
| --- | --- | --- | --- | --- |
| Argentina | La Plata | Instituto Medico Platense | Nicolás Nitti | Nicolás Nitti |
| Argentina | La Plata | Instituto de Diagnóstico de La Plata | Sandra Reyes | Sandra Reyes |
| Argentina | Cordoba | Clínica Universitaria Reina Fabiola | Mario Fernández | Mario Fernández |
| Argentina | Rosario | Hospital Español | Daniel Paolantonio | Daniel Paolantonio |
| Argentina | Cordoba | Sanatorio Aconcagua - Clínica Carrá | Alberto Sampaolesi | Alberto Sampaolesi |
| Argentina | Tucuman | Centro Privado de Cardiología | Pedro Gallardo Galeas | Pedro Gallardo Galeas |
| Belize | Belize City | Cardiology Centre Belize | John Gough | John Gough |
| Bolivia | Santa Cruz de la Sierra | Caja Petrolera de Salud | Erick Hornez | Erick Hornez |
| Bolivia | Santa Cruz de la Sierra | Hospital San Juan de Dios | Luis Alberto Urna Herbas, Jesus Walter Gomez Vargas | Luis Alberto Urna Herbas |
| Brazil | São Paulo | Hospital 9 de Julho | Joao Batista De Freitas Guimaraes | Joao Batista De Freitas Guimaraes |
| Brazil | Teresina | Prontomed | João Francisco de Sousa | Miguel Azevedo |
| Brazil | Sao Joao da Boa Vista | Sancor | Andre Bittar | Andre Bittar |
| Brazil | São Paulo | Hospital Beneficência Portuguesa | José Armando Mangione | José Armando Mangione |
| Brazil | Rio do Sul | HRAV | Gustavo Bravo | Helbok |
| Brazil | Belo Horizonte | Hospital Sao Francisco de Assis | Mauricio C Machado | Mauricio C Machado |
| Brazil | Rio de Janeiro | Marcio Jose Montenegro Da Costa | Márcio Montenegro | Márcio Montenegro |
| Chile | Arica | Unidad de Hemodinamia, Hospital Juan Noé Crevani | Juan Ortega Mosqueira, Augusto Bellet Pacheco | Juan Ortega Mosqueira |
| Chile | Santiago de Chile | Clínica Indisa | Nilo Carvajal Kersanova | Nilo Carvajal Kersanova |
| Chile | Santiago de Chile | Instituto Nacional del Tórax | Jorge Sandoval Berrocal | Jorge Sandoval Berrocal |
| Chile | Temuco | Hospital Temuco | Carlos Olivares | Carlos Olivares |
| Chile | Santiago de Chile | Hospital Clinico Universidad Católica | Gonzalo Martínez | Gonzalo Martínez |
| Chile | Temuco | Hospital Dr. Hernán Henríquez Aravena | Christian Pincetti | Christian Pincetti |
| Chile | Santiago de Chile | San Borja Arriarán | Gabriel Maluenda | Gabriel Maluenda |
| Chile | Osorno | Hospital Base San José de Osorno | Cesar Vargas | Cesar Vargas |
| Chile | Rancagua | Hospital Regional de Rancagua Libertador Bernardo O' Higgins | Marcio Alvarado, Dalila Martinez | Marcio Alvarado |
| Chile | Los Angeles | Hospital Víctor Ríos Ruiz | Anibal Dominguez Lorenzo | Anibal Dominguez Lorenzo |
| Chile | Santiago de Chile | Hospital San Juan de Dios | Angel Puentes Rico | Angel Puentes Rico |
| Chile | Puerto Montt | Hospital Puerto Montt | Víctor Assef | Víctor Assef |
| Chile | Santiago | Hospital Sótero del Rio | Nicolás Veas | Nicolás Veas |
| Chile | La Serena | Hospital San Juan de Dios de la Serena | Alejandro Fleming | Alejandro Fleming |
| Colombia | Ibagué | Instituto Cardiovascular del Tolima | Victor G. Aldana | Victor G. Aldana |
| Colombia | Bogota | Clinica del Country | Alberto Suárez Nitola | Alberto Suárez Nitola |
| Colombia | Bogota | Clínica San Rafael | Jorge Villegas | Jorge Villegas |
| Colombia | Bogota | Clinica Los Nogales | Manuel John Lievano T | Manuel John Lievano T |
| Colombia | Neiva | Clínica Medilaser SA | Pablo Charry | Pablo Charry |
| Colombia | Medellín | Hospital Universitario San Vicente Fundación | Personal | Juan Delgado |
| Colombia | Bogota | Centro Cardiovascular Colombiano Bogotá | William Amaya Ramírez | William Amaya Ramírez |
| Colombia | Cali | Clínica Colombia | Julián Ochoa | Julián Ochoa |
| Colombia | Cali | Angiografía de Occidente S.A. | Director operativo | Jairo Cadena |
| Costa Rica | San José | Hospital México | Luis Gutiérrez Jaikel | Luis Gutiérrez Jaikel |
| Cuba | Havana | CIMEQ | Ronald Aroche Aportela | Ronald Aroche Aportela |
| Ecuador | Quito | Hospital Especialidades Carlos Andrade Marín | Edwin Guzmán | Edwin Guzmán |
| Ecuador | Guayaquil | Hospital Clínica San Francisco | Paulino Quiñonez Rodas | Paulino Quiñonez Rodas |
| Ecuador | Cuenca | Hospital Santa Inés | Ricardo Quizhpe | Ricardo Quizhpe |
| Ecuador | Cuenca | Hospital Jose Carrasco Arteaga | Ricardo Quizhpe | Ricardo Quizhpe |
| Ecuador | Manta | Cardiocentro Manta | Gustavo Hidalgo | Gustavo Hidalgo |
| El Salvador | San Salvador | Circulación | Marco Fuentes | Marco Fuentes |
| Guatemala | Guatemala City | Unidad de Cirugía Cardiovascular de Guatemala (Hospital Roosevelt) | Rodolfo Gutierrez Bartlett | Rodolfo Gutiérrez Bartlett |
| Guatemala | Guatemala City | Unidad de Cirugía Cardiovascular de Guatemala (UNICAR) | Héctor Mora, Alejandro Amado | Hector Mora |
| Honduras | San Pedro Sula | Hospital CemeSa | Francisco Somoza, Josue Ponce | Ramse Amaya |
| Mexico | Mexico City | Privado | Yigar Piña Reyna | Yigar Piña Reyna |
| Mexico | Mexico City | Hospital Mocel | Antón Meneses Bonilla | Antón Meneses Bonilla |
| Mexico | Mexico City | Hospital General Tacuba Issste | Alexis Gómez Anaya | Rodrigo Zenteno Fuentes |
| Mexico | Mexico City | IMSS HG La Raza | Iván González | Fabiola López Madrigal |
| Nicaragua | Managua | Centro Nacional de Cardiología | Daniel Meneses | Daniel Meneses |
| Panama | Panama City | Hospitales Arnulfo Arias Madrid, Paitilla, Nacional, Punta Pacífica, San Fernando, CSS, Santo Tomás | Alfaro Marchena N, Dr. Jaime Dutary, D. Gabriel Frago | Alfaro Marchena N |
| Paraguay | Itauguá | Hospital Nacional de Itaugua | Adrian Ebner, Juan Castellano | Juan Castellano |
| Paraguay | Asunción | Hospital de Clínicas | Gustavo Olmedo, Marcelo Miño | Victor Rojas |
| Paraguay | Asunción | Sanatorio Italiano | Adrian Ebner, Juan Castellano | Victor Rojas |
| Paraguay | Asunción | Centro Médico La Costa | Oscar Bernal, Hugo González | Oscar Bernal |
| Paraguay | Asunción | Sanatorio Britanico | Víctor Adrián Rojas Rodríguez | Victor Rojas |
| Paraguay | Asunción | Sanatorio San Roque | Lorena Villalba, Carlos Benítez | Lorena Villalba |
| Paraguay | Asunción | Instituto Nacional de Cardiología San Jorge | Oscar Bernal, Hugo González | Oscar Bernal |
| Paraguay | Asunción | Instituto de Previsión Social (IPS) | Clotilde Cañete, Victor Rojas | Silvia Vinader |
| Peru | Chiclayo | Hospital Nacional Almanzor Aguinaga Asenjo | William Tito Cornelio Fuster | Piero Custodio Sánchez |
| Peru | Lima | Instituto Nacional Cardiovascular. INCOR. Essalud | César Nicolás Conde Vela | César Nicolás Conde Vela |
| Dominican Republic | Santiago de los Caballeros | Centro de Intervenciones Cardiovasculares (CENICARDIO), Clínica Unión Médica del Norte | Aramis Gómez Belliard | Aramis Gómez Belliard |
| Uruguay | Montevideo | Servicio de Hemodinamia y Cardiología Intervencionista del Hospital Central de las FF.AA. | Daniel Mallo, Ricardo LLuberas | Carolina Artucio |
| Uruguay | Montevideo | Instituto de Cardiología Integral | Ariel Durán | Ariel Durán |
| Uruguay | Salto | Hemodinamia del Litoral | Tomás Dieste, Jorge Mayol | Sebastian Olaizola, Rodrigo Abreu |
| Uruguay | Montevideo | Centro Cardiovascular Universitario del Hospital de Clínicas. | Ariel Durán, Diego Freire | Juan Albistur |
| Uruguay | Montevideo | Instituto Nacional de Cirugía Cardíaca INCC | Cesar Pardiñas | Alex Rocha |
| Uruguay | Montevideo | Cardiocentro - Asociación Española | Ricardo Lluberas, Daniel Mallo | Sebastián Lluberas |
| Uruguay | Montevideo | Centro Cardiológico Americano | Tomás Dieste, Jorge Mayol | Ignacio Batista |
| Uruguay | Montevideo | Instituto de Cardiología Intervencionista de Casa de Galicia | Daniel Mallo, Ricardo LLuberas | Carolina Artucio |
| Venezuela | Caracas | Hospital de Clínicas de Caracas | César Ochoa | César Ochoa |
| Venezuela | Caracas | Clínica Santiago de León | Simón Tovar | Simón Tovar |
| Venezuela | Carabobo | Hemodinamistas de Carabobo | Ramón Cedeño | Lexani Aguilar |
| Venezuela | Maracaibo | Policlínica Amado | Pedro Hidalgo Useche | Pedro Hidalgo Useche |
